# Perception of Animal Abuse among Adolescents: Influence of Social and Demographic Factors

**DOI:** 10.3390/ani14060972

**Published:** 2024-03-21

**Authors:** Laura Estévez-Pérez, Manuel Zumbado, Octavio P. Luzardo, Luis Alberto Henríquez-Hernández

**Affiliations:** 1 Faculty of Veterinary, Universidad de Las Palmas de Gran Canaria, 35413 Arucas, Spain; lauraespe1998@gmail.com; 2 Deontology and Veterinary Legal Unit, Clinical Sciences Department, Universidad de Las Palmas de Gran Canaria, 35001 Las Palmas de Gran Canaria, Spain; manuel.zumbado@ulpgc.es; 3 Toxicology Unit, Clinical Sciences Department, Universidad de Las Palmas de Gran Canaria, 35001 Las Palmas de Gran Canaria, Spain; octavio.perez@ulpgc.es

**Keywords:** animal welfare, animal abuse, survey, adolescent, social factors, hunter, Canary Islands

## Abstract

**Simple Summary:**

The perception of animal abuse is closely linked to each society. Because of its culture, Spain is among the European Union countries with the highest records of animal abuse. The Canary Islands, where this study was carried out, also has very high rates of intentional poisoning of wildlife and abandonment of pets. Care and responsibility towards animals is trained from childhood onwards and is strongly influenced by demographic and social factors. In the current investigation, our aim was to examine the perception of animal abuse among adolescents and to disclose the role of the main social and demographic factors on the perception of animal welfare. To accomplish this objective, we conducted a comprehensive survey targeting individuals aged 14–18 years. We found that males who also had hunting relatives had the least sensitivity to animal abuse.

**Abstract:**

Animal welfare is inherited in each society, shaped by the surrounding environment and upbringing of each individual. This influence becomes particularly significant during adolescence. Due to its cultural context, Spain is among the European Union nations with the highest number of cases of animal abuse. The Canary Islands, the scenario of this study, show the highest rates of intentional poisoning of wildlife and pets’ abandonment. The aim of the present study was to explore the perception of animal welfare among adolescents, studying the influence of the main socio-demographic factors that may condition it. A validated questionnaire on animal abuse was used and distributed to adolescents aged 14–18 years in two public study centers. Animal abuse rates were correlated with socio-demographic variables. In total, 302 subjects answered the questionnaire. The perception of animal welfare was influenced by socio-demographic variables, gender being the most important. The demographic profile of the least responsive adolescent to animal abuse was a male engaged in sports, not owning a dog, and hailing from a family involved in hunting. Awareness should be raised at an early age, promoting artistic activities, encouraging contact with animals and sporting practices that do not generate a lack of empathy for animals.

## 1. Introduction

According to the World Organization for Animal Health (WOAH), an animal is in a good welfare condition if it is healthy, comfortable, well fed, safe, able to express innate forms of behavior and if it does not suffer from unpleasant sensations of pain, fear or distress [1].

The relevance of animal welfare depends on a society’s tolerance of abuse and mistreatment, which is rooted in socio-cultural factors. This explains why animal welfare is more important in northern European countries than in Mediterranean countries [2]. Historically, Spain is a country with a high tolerance for animal abuse. In fact, different forms of mistreatment are even legal and have broad social support. This is the case for bullfighting or cockfighting [3]. It is estimated that there are around 3000 different types of popular festivities in Spain in which animals are used and mistreated. These include cattle, bulls, lambs, horses and different species of birds (ducks) [4]. This cultural background could translate into other forms of animal abuse, including intentional poisoning of domestic animals and wildlife, and animal abandonment [2,5,6]. 

Spain leads Europe in terms of pet abandonment. According to private foundations, 286,000 dogs and cats arrived at animal shelters in our country [7]. If we take into account that there are no data released from some shelters and that there are animals that, once abandoned, live on the streets, we can assume that this number is much higher. According to the European Society of Dog and Animal Welfare (ESDAW), that number could rise to 800,000. There are about one hundred million abandoned pets in Europe [8]. 

The Canary Islands and the Balearic Islands are the Spanish regions where the rate of abandoned animals is higher, and they are among the regions with the highest rates of pet abandonment in Europe. The Canary Islands are a territory highlighted in the reports of the ESDAW, which report a total of 10,000 abandoned dogs. According to the data provided by the main animal shelter on the island of Gran Canaria (the most populated island of the archipelago), a total of 1370 dogs and 635 cats were registered by the end of the year 2022. Similarly, the incidence of poisoning in the Canary Islands is currently one of the highest reported at any region of the European Union [6]. 

There must be certain social and demographic factors that explain these data, taking into account that the Canary Islands have one of the highest levels of social problems in Spain. First, they are one of the autonomous communities with the highest unemployment rates (INE, 2023). Second, according to data from the National Statistics Institute referring to the year 2021 [9], the Canary Islands had the lowest gross disposable household income, with EUR 12,410 per inhabitant (21.5% lower than the national average) [10]. Third, the rate of divorces and marriage annulments keeps the Canary Islands in first place nationally, with a total figure of 4402 divorces per 1000 habitants in 2021, according to data from the statistics service of the General Council of the Judiciary. Fourth, the school failure and dropout rates are one of the highest among the Spanish secondary school. The data show that 11.7% of people between 18 and 24 years old dropped out of school in 2022 [11], the percentage being three times higher among boys. Finally, the country’s demographic pyramid has become asymmetrical in favor of the over-65 age group, whose ageing rate is gradually increasing each year and where births have been at historic lows since 2020 [12]. The ageing index for the Canary Islands is illustrated by the difference between births and deaths registered in 2021: 12,732 births compared to 17,149 deaths [13]. Nowadays, Spain is an ageing country with more pets than children [14]. The official census of animals in the Canary Islands shows a total of 253,126 among dogs, cats, equines and other pets [15]. Dogs are the favorite pet of the Canarian population, where a total of 220,492 dogs are registered, which represents 87% of the total number of animals. However, these data do not take into account those animals that do not have a microchip, mainly cats. 

Despite there being moderate success in animal registration compared to other countries, the data from our country regarding animal welfare and abuse reveal that there must be a cultural basis conditioning the perception of animal mistreatment. Thus, we operate under the following hypothesis: certain socio-demographic variables may influence the perception of animal welfare and abuse within adolescents, considered a particularly sensitive population segment. This is significant as adolescence is a critical period during which numerous behavioral patterns are established, shaping adult attitudes and actions.

The aim of this study was to explore the socio-demographic factors in relation to animal welfare and the perception of animal abuse, in a cohort of adolescents aged 14–18 years belonging to two public educational institutions located on the island of Gran Canaria, for which a specific survey on animal welfare was carried out. 

## 2. Materials and Methods

To assess the perception of adolescents in relation to animal abuse, a modified model of the survey developed by Monzalvo and Torres (2021) [16] was distributed in the guidance departments of the secondary and high schools of Valleseco (CEO Rey Juan Carlos I) and Teror (IES Teror), located in Gran Canaria (Canary Islands, Spain), during September–December 2022. The management of both institutions agreed to participate in the study. The questionnaire was distributed among teachers of 3rd and 4th grade of secondary school, and 1st and 2nd grade of high school. It was the respective teachers who determined when the survey was conducted among their students within the given timeframe. No student refused to participate once informed of the study’s objective and the anonymous nature of the survey. The validated questionnaire includes a total of 18 questions. It was a Likert scale survey with five options: strongly disagree, disagree, neither agree nor disagree, agree and strongly agree; rated with 1, 2, 3, 4 and 5 points, respectively. The survey used in this study included a total of 26 questions (Supplementary File S1) to which a series of descriptive statistics were added (age, gender, type of family, siblings, pet ownership, extracurricular activities or relationship with hunting or fishing, among others). Given the variability of extracurricular activities, these were divided into two main categories: sport activities and artistic activities (which included reinforcement classes or private lessons). Data were collected anonymously complying with all the precepts of the Data Protection Law in force.

Descriptive analyses were conducted for all variables. Means and standard deviation (SD) were calculated for continuous variables. Proportions were calculated for categorical variables. The normality of the data was tested using the Kolmogorov–Smirnov test. Comparisons between groups were performed using parametric (Student *t*-test or ANOVA test) or non-parametric test (Kruskal–Wallis or Mann–Whitney U test). Differences in the categorical variables were tested by the Chi-squared test. We used PASW Statistics v 19.0 (SPSS Inc., Chicago, IL, USA) to manage the database of the study and to perform statistical analyses. Probability levels of <0.05 (two tailed) were considered statistically significant. 

## 3. Results

A total of 302 adolescents between 14 and 17 years old were interviewed about their perception of animal abuse. The main socio-demographic characteristics are summarized in Supplementary File S2. Briefly, 162 individuals (53.6%) were secondary school students, and 164 subjects (54.3%) were male. Most of the individuals lived in a rural environment (90.7%), belonging to a family with married parents (72.8%) and siblings (80.8%). Most of them were involved in after-school activities (67.5%), divided into two categories: sports and artistic activities, including private lessons for reinforcement in this category. Among the sport activities, the most frequently practiced were soccer (n = 42, 13.9% of the total respondents) and gym training (n = 24, 7.9%). Among artistic and reinforcement activities, the majority attended private lessons (n = 33, 10.9% of the total respondents), followed by participation in performing arts activities (n = 23, 7.6%). The majority of individuals lived with a pet (75.5%), dogs being the predominant species (72.8%). A total of 53 (17.5%) and 66 (21.9%) individuals had a relative who was a hunter or fisherman. In order to find out the influence of age on the perception of animal welfare, the sample was segmented according to the school year the individual was attending at the time of the survey. No significant differences were observed in the distribution of the main socio-demographic variables in relation to this segmentation (Supplementary File S2).

### 3.1. Perception of Animal Abuse and Welfare as Reported in Surveys

Table 1 shows the results of the surveys in the whole sample and in relation to the segmentation by academic year. Of the total of 26 questions, 7 showed a significantly different distribution of responses between groups: Q3 (Birds should be kept in cages so that people can admire them; *p* = 0.004), Q4 (If I see that a friend or family member likes to hurt animals, I reprimand them; *p* = 0.029), Q14 (If I couldn’t look after my pet, I would give it up for adoption; *p* = 0.009), Q15 (Street animals are a nuisance and give a bad image to my city; *p* < 0.001), Q17 (Animal fights are fun; *p* = 0.023), Q18 (I would like to have classes on animal care in my high school; *p* = 0.043) and Q21 (At home, they teach who is in charge by hitting my pet; *p* = 0.002).

Viewed in detail, third-year secondary school students showed the highest score (1.86 ± 1.0) referring to Q3 (Birds should be kept in cages so that people can admire them) and the lowest scores (3.72 ± 1.7, and 3.90 ± 1.3) referring to Q4 (If I see that a friend or family member likes to hurt animals, I reprimand them) and Q14 (If I couldn’t look after my pet, I would give it up for adoption), respectively, inferring less sensitivity. On the other hand, first-year high school students showed the highest scores (2.06 ± 1.2, 1.94 ± 1.3 and 1.69 ± 1.1) referring to Q15 (Street animals are a nuisance and give a bad image to my city), Q17 (Animal fights are fun) and Q21 (At home they teach who is in charge by hitting my pet), respectively, inferring less sensitivity. This group of students also showed the lowest scores (1.94 ± 1.3) referring to Q18, inferring less interest.

**Figure 1:**
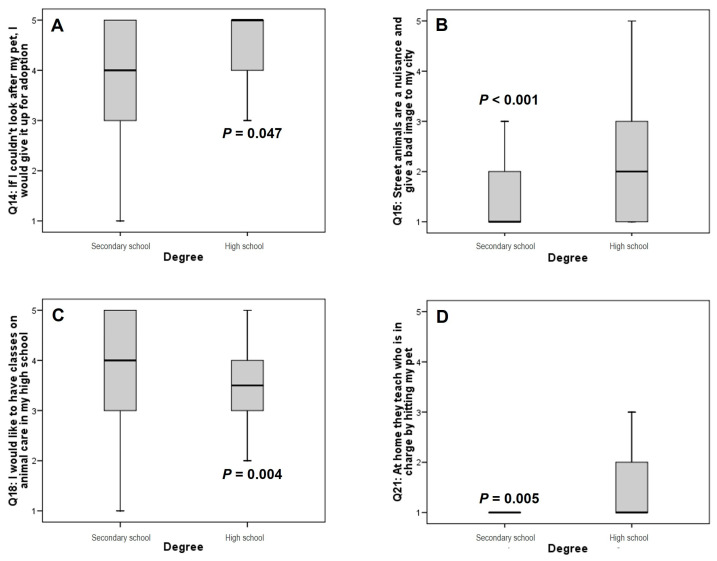
Box plot showing the distribution of answers to the questions Q14 (**A**), Q15 (**B**), Q18 (**C**) and Q21 (**D**) segmented by degree of study. The lines connect the medians, the boxes cover the 25th to 75th percentiles, and the minimal and maximal values are shown by the ends of the bars. *p* values were obtained by ANOVA test.

To better understand these results, the sample was segmented into two groups: secondary school vs. high school students. Questions Q14, Q15, Q18 (I would like to have classes on animal care in my high school) and Q21 maintained their statistical significance (Figure 1). 

From the present results, age seems to be an important factor in the perception of animal welfare, with 14-year-old adolescents (third-year secondary school students) and 16-year-old adolescents (first-year high school students) the least sensitive age groups.

### 3.2. Socio-Demographic Variables Associated to Animal Welfare and Animal Abuse

The associations of socio-demographic variables and the animal welfare scale are shown in Table 2. The gender of the individuals was the factor that showed the most statistically significant differences in terms of number of significant associations (n = 20), followed by the type of afterschool activity (n = 10), and the presence of a family hunter (n = 7). 

**Figure 2:**
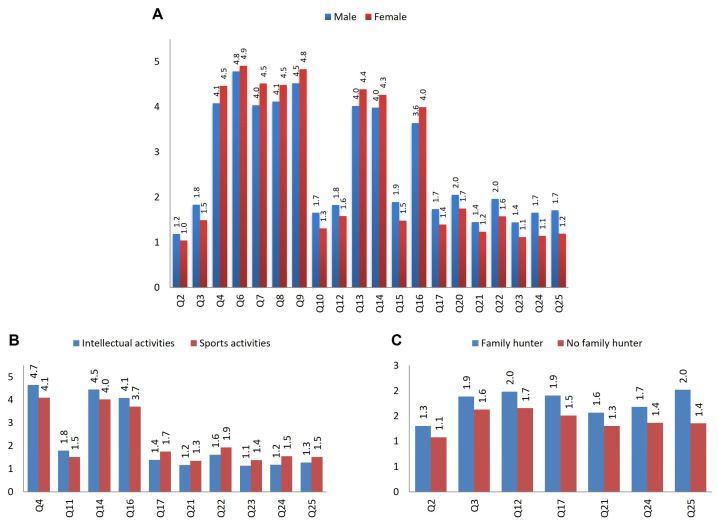
Bar chart showing the significantly different response values in relation to gender (**A**), type of afterschool activity (**B**) and the presence of a family member who hunts (**C**).

In all cases, males showed scores that make them less sensitive to animal abuse (Figure 2A). Females were less likely to agree when asked whether animals suffer less because they are animals (Q2), compared to men (1.0 vs. 1.2, respectively; *p* = 0.009). When asked about the use of violence for animal education (Q12 and Q21), males agreed more than females (1.8 vs. 1.6, *p* = 0.042 and 1.4 vs. 1.2, *p* = 0.019, for Q12 (In my house we use violence, if necessary, to teach the pet what is wrong) and Q21 (At home, they teach who is in charge by hitting my pet), respectively). In all cases, males seem to be more interested in watching dogfighting (Q23), cockfighting (Q24) or bullfighting (Q25) (*p* < 0.001).

A similar pattern was observed with regard to the type of afterschool activity: individuals involved in sports seem to be less sensitive to animal abuse than those involved in intellectual/creative activities (Figure 2B). Thus, the response profile is repeated in relation to some of the questions mentioned above (Q21: 1.3 vs. 1.2, *p* = 0.040; Q23: 1.4 vs. 1.1, *p* = 0.011; Q24: 1.5 vs. 1.2, *p* = 0.001; and Q25: 1.5 vs. 1.3, *p* = 0.049). Questions related to animal care and protection were most sensitively answered by adolescents in intellectual/creative activities: Q4 (If I see that a friend or family member likes to hurt animals, I reprimand them), Q11 (If an animal has a complicated illness, the best thing to do is to get rid of it), Q14 (If I couldn’t look after my pet, I would give it up for adoption) or Q16 (I would like to support an institution where abandoned animals are cared for) (Table 2 and Figure 2B). However, this result has a significant gender bias that warrants caution in its interpretation. Thus, the majority of participants engaged in sports activities were boys (69.3%), while the majority of respondents engaged in intellectual or artistic activities were girls (66.3%) (*p* < 0.001, Chi-squared test).

Although only 17.5% of the sample had a family member who was a hunter, the same pattern as above was repeated: adolescents with this status were less sensitive to animal abuse. This group of individuals was more interested in watching animal fights (Q24 (cockfighting) and Q25 (bullfighting)), even finding it amusing (Q17, Animal fights are fun), considering pets to have less feelings because they are animals (Q2, Animals don’t feel when you hit them because they are animals), and agreeing more with the use of violence for the education of animals (Q12 (In my house we use violence, if necessary, to teach the pet what is wrong) and Q21 (At home, they teach who is in charge by hitting my pet)) (Table 2 and Figure 2C). 

Gender, type of afterschool activity and having a family hunter were all associated with four questions: Q17 (Animal fights are fun), Q21 (At home, they teach who is in charge by hitting my pet), Q24 (I’m curious to see a cock fight) and Q25 (I’m curious to see a bull fight).

While having or not having a pet (of any kind) did not appear as an important factor, dog and cat ownership was associated with Q12 (In my house we use violence, if necessary, to teach the pet what is wrong), Q21 (At home, they teach who is in charge by hitting my pet), Q22 (A dog deserves more care than a cow or a bird) and Q25 (I’m curious to see a bull fight), and to Q15 (Street animals are a nuisance and give a bad image to my city), Q24 (I’m curious to see a cock fight) and Q25 (I’m curious to see a bull fight), respectively (Table 2). Thus, adolescents who owned a dog were less likely to use violence for pets’ education (Q12: 1.6 vs. 1.9; Q21: 1.2 vs. 1.4), and they were less curious to see a bullfight (Q25: 1.6 vs. 1.3). Cat owners were less curious about cockfighting (Q24: 1.3 vs. 1.5) and bullfighting (Q25: 1.3 vs. 1.6). Teenagers with cats were less likely to think that stray animals give the city a bad image (Q15: 1.3 vs. 1.6). 

These findings highlight the importance of this individual profile in the perception of animal welfare: male adolescents who play sports in their spare time, who do not have a dog and who have family members involved in hunting.

Following the strategy of analysis of Monzalvo and Torres [10], the questions were condensed into two groups: “animal care and protection” and “no animal abuse”. The questions were grouped according to the direction of the answers, confirming the profile outlined above with regard to the perception of animal welfare. Thus, higher scores on “Animal care and protection” indicate a greater sensitivity to animal care, while higher scores on “no animal abuse” indicate less sensitivity to animal abuse. Table 3 shows the association of demographic variables and the animal welfare scale segmented in these two groups. Gender was associated with both groups (*p* < 0.001), as well as the type of afterschool activity (*p* = 0.018 and *p* = 0.012, respectively). Having a relative who was a hunter was associated with the group of questions grouped as “no animal abuse” (*p* < 0.001). In addition, it is interesting to note that the greater the number of species kept as pets, the greater the animal care and protection (46.9, 47.8 and 45.8 for 1, 2 and ≥3 different species, respectively; *p* = 0.046).

## 4. Discussion

Humans have an innate affinity with the living world that leads us to interact and form emotional bonds with other life forms [17], giving rise to the human–animal bond, defined by the American Veterinary Medical Association (AVMA) as “a dynamic and mutually beneficial relationship between humans and other animals that is influenced by behaviors essential to the health and well-being of both” [18]. This type of bond is particularly important for children and young people. In that sense, Collins and McNicholas (1998) found that children consider their pets to be close family members, not only because they live in the same house, but also because of the functions they perform [19]. In fact, children who develop a bond with their pets have higher scores in empathy, self-esteem and self-knowledge than those who do not have pets [20,21,22]. In addition, pet ownership promotes the development of trust, responsibility and compassion, among other qualities [23]. For this reason, a possible explanation is found for the data obtained in the analysis of the adolescent study, in which those who did not have pets were more likely to use violence to train their supposed pet than those who had animals at home. Understanding pets as members of the family [22], the bond that children develop with their pets is particularly strong in families with multiple dysfunctional factors, such as social disadvantage, poverty or poor parental education, as well as crime and substance abuse [24]. In fact, many authors show that those children who experience family or animal violence are most likely to have been exposed to some additional type of abuse, suggesting the presence of a violent family environment [25].

Cruelty to animals is defined differently according to the environment in which the owners live (rural vs. urban) [26]. In this way, animals in rural areas are attributed mainly practical functions [27] rather than companionship and affection, and their ability to feel pain, hunger or sadness is often ignored. This observation agrees with the results of our study, although the result should be interpreted with caution due to the bias produced by the fact that more than 90% of the sample lived in a rural environment.

Spain is one of the European Union countries with the highest number of hunters. According to the Federation of Associations for Hunting and Conservation of the European Union [28], our country registered 980,000 hunters in 2010, making it the second country with the most hunters after France [29]. This means that 2% of the Spanish population practices hunting, with men being the main practitioners [30]. However, of this total number of hunters, only half were registered with the Spanish Hunting Federation in the same year [31]. Furthermore, when hunting with dogs, most of them have at least one animal, although it is common to have more than one [32]. Moreover, according to the latest report published by the Affinity Foundation (2022) on dogs and cats abandoned in Spain during 2017, the end of the hunting season was the second cause of pet abandonment in Spain, reinforcing the idea that animals in rural areas have a purely practical function [33].

Children in the study who had hunting relatives were particularly interested in watching an animal fight (Q17: Animal fights are fun), whether it was a dog fight (Q23: I am curious to see a dog fight), a cock fight (Q24: I am curious to see a cockfight) and/or a bullfight (Q25: I am curious to see a bull fight), a factor that is likely to be explained by the festive environment surrounding hunting and the normalized violence of such an act [34]. Relationships in the rural world are much closer than in the city [35]. This sharing is particularly worrying given the explicit peer approval of this type of violence and the rewards for those who perpetrate it [34].

Social learning plays a role in the mistreatment of animals by children and adolescents, especially when these behaviors are perpetrated by important figures in their lives [25]. Furthermore, in the study ‘Rural and urban differences in the commission of animal cruelty’ (2005), rural respondents were mainly affected by witnessing family members mistreating animals, whereas urban respondents learned about cruelty from family and friends [26]. This could be a reason why in Question Q20 (It’s normal for my grandparents to raise their pets by beating them, and I can’t do anything about it because they are from another era), higher scores—agreement—were obtained from young people from rural areas than from those living in urban areas. Something similar happened with Question Q4 (If I see a relative or a friend beating animals, I draw their attention to it). 

It has been described that violence against legally protected animals, such as hunting, is often accompanied by other unauthorized expressions of violence because of the festive and tolerant atmosphere surrounding the violence [36]. According to research by Clifton Flynn (2002) among young people, males who hunted were twice as likely as non-hunters to have committed acts of animal cruelty against stray and/or wild animals [34]. This was confirmed in the present study with Question Q15 (Stray animals give my town a bad image), where minors with hunting relatives scored higher than those without.

The present results suggest that patterns of animal abuse are inherited or learned, and the determining factor is the sociodemographic environment in which the child is surrounded, which defines a specific individual profile: male, without a dog, living in rural areas, with family members involved in hunting or fishing. The results of this study are in line with previous literature in the sense that adolescents at the age of 14 are less sensitive to animal welfare than those at the age of 16, a factor that can be explained by age as a modulating variable of empathy [37].

Empathy is also modulated by gender. According to the literature, women are more empathetic than men, both towards humans and animals [38], while men are more likely to be violent towards animals [39]. There are several studies that link gender-based violence to cruelty towards animals [24,40,41], stating that aggression towards animals is related with aggression towards humans [42]. A positive correlation was observed between domestic violence and negative interactions with the household pet. The use of these types of violence has been confirmed by various interviews with women victims of gender-based violence in different shelters, most of whom stated that their abuser had previously threatened and/or beaten their pet [43,44,45]. This is because animal abuse ‘socializes’ the perpetrator with violence, symbolizing the crossing of a barrier, and once the animal has been abused, there are fewer inhibitions, making acts of cruelty towards other family members more likely [46]. Taking together, animal abuse is a useful indicator of gender-based violence, which is why countries such as the United States, the United Kingdom and Australia have established monitoring protocols to alert authorities to animal abuse which may indicate the potential for gender-based violence [47]. In that sense, we agree with the published data, according the results obtained from Questions Q12 (In my house we use violence, if necessary, to teach the pet what is wrong) and Q21 (At home, they teach who is in charge by hitting my pet). Within the narrative of hunting, where the ‘predator is played’, a patriarchal culture is favored in which masculinity is defined as aggressive, powerful and violent [45]. In a study of abusive couples, 52% who abused their pets also hunted, compared to only 11% who had pets and did not engage in this form of abuse [36]. 

An important finding during this study was the modulation of the perception of animal welfare/animal abuse according to the type of extracurricular activity: those engaged in sport were much less sensitive to animal abuse than those engaged in intellectual or creative activities (Figure 2B, Questions Q21 (At home, they teach who is in charge by hitting my pet), Q23 (I’m curious to see a dog fight), Q24 (I’m curious to see a cock fight) and Q25, (I’m curious to see a bull fight)). These results reinforce the conclusions obtained after carrying out the support macroprocess: art is a potential generator of empathic processes, as it raises people’s awareness in a critical way, leading them to reflect on social realities, such as animal abuse, and consequently, to develop responses and solutions [48,49].

Finally, our results have shown a certain interest in receiving animal welfare education (Q18). For this reason, we propose the implementation of a School Prevention of Animal Abuse program, aimed primarily at children between 14 and 16 years of age, in which it is proposed to carry out an animal welfare project that contributes to putting into practice their knowledge and skills in this area, as well as reflecting on their treatment of animals [50]. In the end, for humane education to be successful, it must be developed on an emotional level rather than along rational lines.

## 5. Conclusions

The perception of animal welfare was influenced by socio-demographic variables, with gender being the most important. The demographic profile of the least responsive adolescent to animal abuse was a male engaged in sports, not owning a dog, and hailing from a family involved in hunting. Awareness should be raised at an early age, promoting artistic activities, encouraging contact with animals and sporting practices that do not generate a lack of empathy for animals.

## Figures and Tables

**Table 1:** Distribution (mean ± standard deviation (SD)) of the variables of the animal welfare scale in the whole population of adolescents and segmented by academic year.

	Whole Sample	Secondary School			High School		*p* Value *
	(n = 302)	3rd Degree (n = 88)	3rd Degree * (n = 18)	4th Degree (n = 56)	1st Degree (n = 65)	2nd Degree (n = 75)	
Variable †	Mean ± SD	Mean ± SD	Mean ± SD	Mean ± SD	Mean ± SD	Mean ± SD	
Q1	1.05 ± 0.3	1.08 ± 0.3	1.00 ± 0.0	1.05 ± 0.3	1.02 ± 0.1	1.04 ± 0.2	0.532
Q2	1.12 ± 0.5	1.14 ± 0.6	1.06 ± 0.2	1.18 ± 0.7	1.17 ± 0.6	1.01 ± 0.1	0.294
**Q3**	1.67 ± 0.8	1.86 ± 1.0	1.67 ± 0.8	1.59 ± 0.7	1.80 ± 0.8	1.40 ± 0.7	**0.004**
**Q4**	4.25 ± 1.3	4.08 ± 1.5	3.72 ± 1.7	4.57 ± 0.9	4.11 ± 1.4	4.48 ± 1.2	**0.029**
Q5	4.73 ± 0.6	4.67 ± 0.7	4.67 ± 1.0	4.80 ± 0.5	4.74 ± 0.5	4.75 ± 0.6	0.765
Q6	4.84 ± 0.5	4.81 ± 0.6	4.94 ± 0.2	4.88 ± 0.5	4.77 ± 0.5	4.89 ± 0.3	0.397
Q7	4.25 ± 0.9	4.30 ± 0.9	4.22 ± 0.8	4.34 ± 0.9	4.22 ± 0.9	4.19 ± 0.9	0.866
Q8	4.28 ± 0.9	4.30 ± 0.9	4.22 ± 1.1	4.36 ± 0.8	4.23 ± 1.0	4.28 ± 0.8	0.954
Q9	4.67 ± 0.8	4.51 ± 0.9	4.78 ± 0.5	4.79 ± 0.7	4.63 ± 0.8	4.76 ± 0.5	0.150
Q10	1.49 ± 0.8	1.56 ± 1.0	1.39 ± 0.6	1.27 ± 0.5	1.58 ± 0.8	1.53 ± 0.9	0.217
Q11	1.66 ± 1.0	1.68 ± 1.0	1.50 ± 0.9	1.64 ± 1.1	1.68 ± 1.0	1.67 ± 0.9	0.967
Q12	1.71 ± 1.0	1.73 ± 1.1	1.83 ± 1.3	1.61 ± 0.9	1.89 ± 1.2	1.59 ± 0.9	0.422
Q13	4.19 ± 1.0	4.32 ± 0.9	4.06 ± 0.9	4.32 ± 0.8	4.00 ± 1.2	4.13 ± 1.0	0.231
**Q14**	4.11 ± 1.1	3.90 ± 1.3	4.00 ± 1.3	4.14 ± 1.1	3.97 ± 1.1	4.49 ± 0.7	**0.009**
**Q15**	1.70 ± 1.0	1.47 ± 0.8	1.50 ± 0.9	1.45 ± 0.8	2.06 ± 1.2	1.91 ± 1.1	**<0.001**
Q16	3.80 ± 1.3	4.03 ± 1.2	3.44 ± 1.2	3.66 ± 1.4	3.65 ± 1.3	3.85 ± 1.2	0.193
**Q17**	1.58 ± 1.1	1.45 ± 1.0	1.33 ± 1.0	1.66 ± 1.2	1.94 ± 1.3	1.40 ± 0.9	**0.023**
**Q18**	3.69 ± 1.2	3.91 ± 1.2	3.83 ± 1.2	3.82 ± 1.2	3.35 ± 1.1	3.59 ± 1.1	**0.043**
Q19	3.00 ± 1.0	3.16 ± 1.1	3.06 ± 1.1	2.89 ± 1.0	2.97 ± 0.9	2.92 ± 1.0	0.500
Q20	1.91 ± 1.1	1.85 ± 1.1	1.89 ± 1.2	1.84 ± 1.1	2.02 ± 1.1	1.95 ± 1.1	0.883
**Q21**	1.35 ± 0.8	1.25 ± 0.7	1.06 ± 0.2	1.25 ± 0.8	1.69 ± 1.1	1.31 ± 0.7	**0.002**
Q22	1.78 ± 1.1	1.69 ± 1.0	1.56 ± 0.8	1.98 ± 1.2	1.94 ± 1.2	1.65 ± 0.9	0.204
Q23	1.29 ± 0.7	1.26 ± 0.7	1.11 ± 0.5	1.25 ± 0.8	1.43 ± 0.8	1.28 ± 0.7	0.453
Q24	1.42 ± 0.9	1.39 ± 0.9	1.11 ± 0.5	1.34 ± 0.9	1.62 ± 1.1	1.41 ± 0.9	0.252
Q25	1.47 ± 1.0	1.49 ± 1.0	1.44 ± 0.9	1.41 ± 1.0	1.65 ± 1.1	1.35 ± 0.8	0.478
Q26	1.34 ± 0.8	1.41 ± 0.8	1.06 ± 0.2	1.25 ± 0.7	1.43 ± 0.8	1.31 ± 0.8	0.283

Q1: When I have a pet at home and we no longer want it, the best thing to do is to leave it on the street. Q2: Animals don’t feel when you hit them because they are animals. **Q3**: Birds should be kept in cages so that people can admire them. **Q4**: If I see that a friend or family member likes to hurt animals, I reprimand them. Q5: When I have a pet, I like to be responsible and take care of it. Q6: In my house, we treat animals well. Q7: I like to give water or food to animals in the street. Q8: If I see an animal being mistreated, it is my duty to defend it. Q9: I have been taught at home that I should respect animals. Q10: Animals are only good for people’s amusement. Q11: If an animal has a complicated illness, the best thing to do is to get rid of it. Q12: In my house we use violence, if necessary, to teach the pet what is wrong. Q13: When I see an animal in the street, I would like to help it. **Q14**: If I couldn’t look after my pet, I would give it up for adoption. **Q15**: Street animals are a nuisance and give a bad image to my city. Q16: I would like to support an institution where abandoned animals are cared for. **Q17**: Animal fights are fun. **Q18**: I would like to have classes on animal care in my high school. Q19: I have the feeling that animals are mistreated. Q20: It is normal that my grandparents taught the animals by hitting them and I can’t do anything about it because they are from another era. **Q21**: At home, they teach who is in charge by hitting my pet. Q22: A dog deserves more care than a cow or a bird. Q23: I’m curious to see a dog fight. Q24: I’m curious to see a cock fight. Q25: I’m curious to see a bull fight. Q26: I would have stuffed animals at home. * ANOVA test. † All variables were normally distributed according to Kolmogorov–Smirnoff test (*p* < 0.001 in all cases). Questions (Q) highlighted in bold indicate statistically significant results.

**Table 2:** Association of demographic variables and the animal welfare scale.

	Gender *	Habitat *	Family Situation **	Siblings *	Afterschool Activities *	Type of Activity *	Pets *	No of Pets **	No of Species **	Cats *	Dogs *	Family Hunter *	Family Fisherman *	N
Q1	ns	ns	0.018	ns	ns	ns	ns	ns	ns	ns	ns	ns	ns	1
Q2	0.009	ns	ns	ns	0.032	ns	ns	ns	ns	ns	ns	0.003	ns	3
Q3	<0.001	ns	ns	ns	ns	ns	ns	ns	ns	ns	ns	0.037	ns	2
Q4	0.011	ns	0.005	ns	ns	0.001	ns	ns	ns	ns	ns	ns	ns	3
Q5	ns	ns	ns	ns	ns	ns	ns	ns	ns	ns	ns	ns	ns	0
Q6	0.023	ns	ns	ns	ns	ns	ns	ns	ns	ns	ns	ns	ns	1
Q7	<0.001	ns	ns	ns	ns	ns	ns	0.029	0.005	ns	ns	ns	ns	3
Q8	<0.001	ns	ns	ns	ns	ns	ns	ns	0.011	ns	ns	ns	ns	2
Q9	<0.001	ns	ns	ns	ns	ns	ns	ns	ns	ns	ns	ns	ns	1
Q10	<0.001	ns	ns	ns	ns	ns	ns	ns	ns	ns	ns	ns	ns	1
Q11	ns	ns	ns	ns	ns	0.038	ns	ns	ns	ns	ns	ns	ns	1
Q12	0.042	ns	ns	ns	ns	ns	0.003	ns	ns	ns	0.038	0.037	ns	4
Q13	0.001	ns	ns	ns	ns	ns	0.011	ns	ns	ns	ns	ns	ns	2
Q14	0.024	ns	0.017	ns	ns	0.002	ns	ns	ns	ns	ns	ns	ns	3
Q15	<0.001	ns	ns	ns	ns	ns	ns	ns	0.014	0.001	ns	ns	ns	3
Q16	0.017	ns	ns	ns	ns	0.030	ns	ns	ns	ns	ns	ns	ns	2
Q17	0.006	ns	ns	ns	ns	0.011	ns	ns	ns	ns	ns	0.016	ns	3
Q18	ns	ns	ns	ns	ns	ns	ns	ns	ns	ns	ns	ns	ns	0
Q19	ns	ns	ns	ns	ns	ns	ns	ns	ns	ns	ns	ns	ns	0
Q20	0.016	ns	ns	ns	ns	ns	ns	ns	ns	ns	ns	ns	ns	1
Q21	0.019	ns	ns	0.031	0.042	0.040	ns	ns	ns	ns	0.046	0.029	ns	6
Q22	0.001	ns	ns	ns	ns	0.023	ns	ns	ns	ns	0.019	ns	ns	3
Q23	<0.001	ns	ns	ns	ns	0.011	ns	ns	ns	ns	ns	ns	ns	2
Q24	<0.001	ns	ns	ns	ns	0.001	ns	ns	ns	0.045	ns	0.024	ns	4
Q25	<0.001	ns	ns	ns	ns	0.049	ns	ns	ns	0.040	0.037	<0.001	ns	5
Q26	ns	ns	ns	ns	ns	ns	ns	ns	ns	ns	ns	ns	ns	0
N	20 (76.9)	0	3(11.5)	1 (3.8)	2 (7.7)	10 (38.5)	2 (7.7)	1 (3.8)	3 (11.5)	3 (11.5)	4 (15.4)	7 (26.9)	0	

Abbreviation: ns, non-significant; N, number of significant associations. * Student *t*-test. ** ANOVA test.

**Table 3:** Distribution of demographic variables significantly associated with animal care and protection and no animal abuse. Numbers show the mean and standard deviation. Significant *p* values are included.

	Animal Care and Protection	*p* Value	No Animal Abuse	*p* Value
Gender *		<0.001		<0.001
Male	46.5 ± 5.8		22.6 ± 6.4	
Female	48.9 ± 5.2		18.6 ± 4.5	
Type of activity *†		0.018		0.012
Intellectual	49.0 ± 4.6		19.4 ± 4.2	
Sports	47.3 ± 5.6		21.3 ± 6.6	
Number of species **		0.046		ns
1	46.9 ± 6.2		—	
2	47.8 ± 4.6		—	
≥3	48.5 ± 5.4		—	
Cats *		ns		0.046
Yes	—		19.8 ± 4.9	
No	—		21.4 ± 6.7	
Family hunter *		ns		<0.001
Yes	—		23.7 ± 7.8	
No	—		20.1 ± 5.3	

Abbreviation: ns, non-significant. Animal care protection represents the sum of Questions 4, 5, 6, 7, 8, 9, 13, 14, 16, 18, 19 and 22, according to a modification from Montalvo-Curriel et al. [16]. No animal abuse represents the sum of Questions 1, 2, 3, 10, 11, 12, 15, 17, 20, 21, 23, 24, 25 and 26 according to a modification from Montalvo-Curriel et al. [16]. * Student *t*-test. ** ANOVA test. † Intellectual afterschool activities include art performance and support classes; sports include football, swimming, athletics, gymnastics and horse riding, among others.

## Data Availability

Data are contained within the article and Supplementary Materials.

## Supplementary Materials

The following supporting information can be downloaded at: https://www.mdpi.com/article/10.3390/ani14060972/s1, Supplementary File S1: Survey used in this study; Supplementary File S2: Demographic characteristics of the study population, in the whole sample and segmented according to level of education.
