# Supplementary material for: Perception of Animal Abuse among Adolescents: Influence of Social and Demographic Factors
Source: Animals (Basel). 2024 Mar 21;14(6):972. doi: 10.3390/ani14060972 (PMC10967294; doi:10.3390/ani14060972)
Supplement: Supplementary file 1 [file animals-14-00972-s001.zip › Supplementary File S1.pdf]

ENCUESTA

NIVEL DE EDUCACIÓN EMOCIONAL EN RELACIÓN A LA PREVENCIÓN DEL MALTRATO ANIMAL

INFORMACIÓN GENERAL

La presente encuesta está destinada a evaluar el nivel de educación emocional en relación a la prevención del maltrato animal. Forma parte de un Trabajo de Fin de Grado de la Universidad de Las Palmas de Gran Canaria (Facultad de Veterinaria). Está destinada a adolescentes comprendidos entre los 14 y los 17 años: estudiantes de 3º y 4º de ESO, y de 1º y 2º de Bachillerato. Es totalmente ANÓNIMA y VOLUNTARIA.

Te pedimos, por favor, que leas atentamente las preguntas y respondas con libertad y sinceridad. Responde con un 1, si estás totalmente de desacuerdo, en una escala que llega hasta el 5, si estás totalmente de acuerdo con lo que se está preguntando.

Muchísimas gracias por tu colaboración.

CUESTIONARIO

Datos demográficos: rellena los siguientes datos según corresponda

Sexo biológico (hombre/mujer):
Edad (años):
Municipio en el que vives:
Curso:
Entorno en el que vives (rural/urbano):
Tipo de familia (padres casados/padres separados/otros):
Hermanos (sí o no):
Si tienes hermanos, decir la posición que ocupas:
Actividades extraescolares (sí o no):
Si realizas actividades extraescolares, di cuál:
Tienes mascota (sí o no):
Si tienes mascota, decir cuántas:
Si tienes mascota, decir cuáles (perro, gato, conejo, pájaros, reptiles):
¿Algún familiar es cazador? (sí o no):
¿Algún familiar es pescador? (sí o no):

ENCUESTA DE PREVENCIÓN DE MALTRATO ANIMAL

Contesta a las siguientes preguntas aunque no tengas mascota.

	Totalmente en desacuerdo (1)	En desacuerdo (2)	Ni de acuerdo ni en desacuerdo (3)	De acuerdo (4)	Totalmente de acuerdo (5)
Cuando tengo una mascota en casa y ya no la queremos, lo mejor es dejarla en la calle					
Los animales no sienten cuando los golpeas porque son animales					
Los pájaros deben estar en jaulas para que la gente los pueda admirar					
Si veo que a un amigo o familiar le gusta hacer daño a los animales, le reprendo					
Cuando tengo una mascota me gusta ser responsable y cuidarla					
En mi casa tratamos bien a los animales					
Me gustaría darles agua o comida a los animales de la calle					
Si veo que maltratan a un animal, es mi deber defenderlo					
En casa me han enseñado que debo respetar a los animales					
Los animales solo sirven para divertir a las personas					
Si un animal tiene una enfermedad complicada, lo mejor es deshacerse de él					
En mi casa usamos la violencia, si hace falta, para enseñar a la mascota lo que está mal					
Cuando veo un animal en la calle, quisiera ayudarlo					
Si no pudiera cuidar a mi mascota, la daría en adopción					
Los animales de la calle molestan y dan mala imagen a mi ciudad					
Me gustaría apoyar a una institución donde cuiden					

animales abandonados					
Las peleas entre animales son divertidas					
Me gustaría que se dieran clases sobre cuidado de los animales en mi instituto					
Tengo la sensación de que los animales están maltratados					
Es normal que mis abuelos enseñaran a los animales pegándoles y no puedo hacer nada porque son de otra época					
En casa me enseñan quién manda pegándole a mi mascota					
Un perro se merece más cuidados que una vaca o un pájaro					
Tengo curiosidad por ver una pelea de perros					
Tengo curiosidad por ver una pelea de gallos					
Tengo curiosidad por ver una corrida de toros					
Tendría animales disecados en casa					

Muchísimas gracias por tu participación. Dale el cuestionario a tu profesor(a). Cuando terminemos el trabajo de investigación, lo remitiremos a tu instituto para que puedas leer las conclusiones que salgan de este trabajo de investigación.
