# Supplementary material for: Perception of Animal Abuse among Adolescents: Influence of Social and Demographic Factors
Source: Animals (Basel). 2024 Mar 21;14(6):972. doi: 10.3390/ani14060972 (PMC10967294; doi:10.3390/ani14060972)
Supplement: Supplementary file 1 [file animals-14-00972-s001.zip › Supplementary File S2.pdf]

Supplementary File S2. Demographic characteristics of adolescents studied, in the whole series and segmented according to year of study.

	**Whole sample**	**Secondary school**			**High school**		**P value#**
		3rd degree	3rd degree*	4th degree	1st degree	2nd degree	
	(n = 302)	(n = 88)	(n = 18)	(n = 56)	(n = 65)	(n = 75)	
**Variable**	n (%)	n (%)	n (%)	n (%)	n (%)	n (%)	
Gender							0.467
Male	164 (54.3)	49 (55.7)	11 (61.1)	26 (46.4)	40 (61.5)	38 (50.7)	
Female	138 (45.7)	39 (44.3)	7 (38.9)	30 (53.6)	25 (38.5)	37 (49.3)	
Age (mean ± SD)	15.5 ± 1.2	14.2 ± 0.5	14.8 ± 0.4	15.0 ± 0.6	16.0 ± 0.5	17.0 ± 0.3	< 0.001†
Level of education							—
Secundary school	162 (53.6)	NA	NA	NA	NA	NA	
High school	140 (46.4)	NA	NA	NA	NA	NA	
Habitat							0.242
Rural	274 (90.7)	79 (89.8)	18 (100)	54 (96.4)	57 (87.7)	66 (88.0)	
Urban	92 (9.3)	9 (10.2)	0	2 (3.6)	8 (12.3)	9 (12.0)	
Family situation							0.951
Married parents	220 (72.8)	63 (71.6)	13 (72.2)	40 (71.4)	47 (72.3)	57 (76.0)	
Divorced parents	64 (21.2)	18 (20.5)	3 (16.7)	13 (23.2)	15 (23.1)	15 (20.0)	
Others	18 (6.0)	7 (8.0)	2 (11.1)	3 (5.4)	3 (4.6)	3 (4.0)	
Siblings (yes)	244 (80.8)	71 (80.7)	16 (88.9)	45 (80.4)	52 (80.0)	60 (80.0)	0.935
Afterscholl activities (yes)	204 (67.5)	60 (68.2)	5 (27.8)	38 (67.9)	46 (70.8)	55 (73.3)	0.006
Type of activity**							0.039
Intellectual	80 (39.2)	28 (46.7)	2 (40.0)	18 (47.4)	9 (19.6)	23 (41.8)	
Sports	124 (60.8)	32 (53.3)	3 (60.0)	20 (52.6)	37 (80.4)	32 (58.2)	
Pets (yes)	228 (75.5)	73 (83.0)	15 (83.3)	42 (75.0)	47 (72.3)	51 (68.0)	0.208
Number of pets							0.807
1	83 (36.4)	27 (37.0)	5 (33.3)	13 (31.0)	19 (40.4)	19 (37.3)	
2	49 (21.5)	18 (24.7)	2 (13.3)	9 (21.4)	11 (23.4)	9 (17.6)	
3 – 5	63 (27.6)	22 (30.1)	4 (26.7)	12 (28.6)	12 (25.5)	13 (25.5)	
≥6	33 (14.5)	6 (8.2)	4 (26.7)	8 (19.0)	5 (10.6)	10 (19.6)	

Number of species							0.058
1	134 (58.8)	44 (60.3)	8 (53.3)	22 (52.4)	29 (61.7)	31 (60.8)	
2	56 (24.6)	20 (27.4)	7 (46.7)	14 (33.3)	10 (21.3)	12 (23.5)	
≥3	38 (16.7)	9 (12.3)	0	6 (14.3)	8 (17.0)	8 (15.7)	
Cats (yes)	82 (36.0)	21 (28.8)	8 (53.3)	17 (40.5)	20 (42.6)	16 (31.4)	0.255
Dogs (yes)	166 (72.8)	49 (67.1)	12 (80.0)	30 (71.4)	35 (74.5)	40 (78.4)	0.644
Family hunter (yes)	53 (17.5)	13 (14.8)	3 (16.7)	6 (10.7)	19 (29.2)	12 (16.0)	0.074
Family fisherman (yes)	66 (21.9)	22 (25.0)	4 (22.2)	9 (16.1)	14 (21.5)	17 (22.7)	0.801

Abbreviations: SD, standard deviation; NA, not applicable.

*Subgroup of students with adapted syllabus mainly due to learning difficulty.

**Intellectual afterschool activities include art performance and support classes; sports include soccer, swimming, athletics, gymnastics, and horse riding, among others.

#Chi square test.

†Kruskal-Wallis test.
